# The need to consider auxiliary assumptions in preregistration practices

**DOI:** 10.3389/fpsyg.2025.1633990

**Published:** 2025-10-15

**Authors:** Tom St Quinton, David Trafimow

**Affiliations:** ^1^School of Humanities and Social Sciences, Leeds Beckett University, Leeds, United Kingdom; ^2^Department of Psychology, New Mexico State University, Las Cruces, NM, United States

**Keywords:** auxiliary assumptions, preregistration, questionable research practices, replication, Open Science

## Abstract

The importance of preregistration has gained recent traction in psychology. To reduce questionable research practices and improve the credibility of research findings, researchers preregister important details before commencing with data collection. However, current preregistration practices miss an important issue when it comes to evaluating predictions. That is because predictions depend not only on theoretical terms but also auxiliary assumptions. Auxiliary assumptions traverse the distance from nonobservational theoretical terms to observational terms at the level of the empirical hypotheses. Because the credibility of study findings depends on the appraisal of auxiliary assumptions, these assumptions should, at least, be considered in preregistration practices. In this paper we outline the need to consider auxiliary assumptions during preregistration, the benefits of doing so, and how current practices can be amended to accommodate them. If the need for researchers to preregister continues to increase and the belief is that doing so will increase the credibility of psychological research, we believe auxiliary assumptions should become part of these practices.

## Introduction

Whether intentional or not, researchers engaging in questionable research practices (QRPs) can impede the replication of study findings (Baker and Penny, 2016; Open Science Collaboration, 2015; Shrout and Rodgers, 2018). To circumvent such problems, psychology has embraced the adoption of preregistration whereby researchers are transparent about important study details prior to data collection (e.g., rationale, hypotheses, methods, analysis plan). Despite the uptake of preregistration in psychology, an alternative view is that credibility issues originate from poor use of theory and evaluation (e.g., Oberauer and Lewandowsky, 2019; Szollosi and Donkin, 2021). We present in this paper such a theoretical issue. Specifically, we suggest that researchers should pay greater attention to the auxiliary assumptions associated with their predictions. Auxiliary assumptions bridge the gap between nonobservational theoretical terms and observable empirical tests (Lakatos, 1976). Without considering auxiliary assumptions, researchers cannot be confident that their empirical victories and defeats reflect scientific reality. Although preregistration practices currently do little to account for auxiliary assumptions, we believe opportunities exist for their inclusion.

To make our point, we briefly introduce preregistration and its use in psychology. Subsequently, we introduce auxiliary assumptions and the importance of considering these assumptions. Following this, we discuss the importance of auxiliary assumptions in the context of preregistration. We hope that this paper informs readers about the importance of auxiliary assumptions and subsequently leads to a greater consideration of these assumptions in preregistration practices.

## Preregistration and theory

Replication failures in psychology indicate that many findings may be incorrect (Open Science Collaboration, 2015; Nosek et al., 2022; Pashler and Wagenmakers, 2012; Shrout and Rodgers, 2018). QRPs, such as cherry-picking results, *p*-hacking, hypothesizing after the results are known (HARKing; Kerr, 1998), and reporting bias (Schimmack, 2020), can contribute to failed replications. With many psychology researchers admitting to having performed QRPs (John et al., 2012), a solution has been offered through preregistration. Briefly, preregistration involves researchers making available key study details prior to commencing with data collection. Researchers can deposit information about their rationale, methods, design, hypotheses, and analytic plans on various online platforms (e.g., Open Science Framework, AsPredicted, BMJ Open). Amongst other benefits, preregistration is stated to decrease publication bias, overestimated effects, and type 2 errors (Lakens, 2019; Schimmack, 2020; Wicherts et al., 2016), and improve replications (Freese, 2007) and psychological science more generally (Nosek et al., 2018; Vazire, 2018).

Although preregistration can, to some degree, nullify QRPs, an alternative view is that credibility and replication issues are down to theoretical problems (e.g., Fiedler, 2017; Klein, 2014; Muthukrishna and Henrich, 2019; Oberauer and Lewandowsky, 2019; Reber, 2016; Szollosi and Donkin, 2021). For example, Szollosi and Donkin (2021) believe psychological theories are too malleable; Muthukrishna and Henrich (2019) suggest psychology lacks an overarching theoretical framework; and Reber (2016) suggests psychologists ask the wrong questions. In taking this position, implementing methodological reforms, such as preregistration practices, would have limited effect on the validity of psychological research. Theoretical improvements may in fact render methodological reforms unnecessary (Szollosi and Donkin, 2021).

We take the view that the use of theory is a major concern in psychological research. Specifically, we believe that greater attention should be given to the auxiliary assumptions associated with a prediction. However, we believe that preregistration platforms could be amended to cater for this theoretical issue. Providing researchers the opportunity to detail auxiliary assumptions could improve the quality and credibility of psychological research. We will now introduce auxiliary assumptions before outlining their potential role in preregistration practices.

## Auxiliary assumptions

Popper (1959) suggested that scientists should aim to falsify theories. We can see this in syllogism 1:

### Syllogism 1

Premise 1: if the theory is true, then the finding should be true.

Premise 2: the finding is not true.

Conclusion: therefore, the theory is not true.

Duhem (1954) and Lakatos (1976) made some important observations about this syllogism. Rather than theory alone, they suggested that predictions require the consideration of auxiliary assumptions. Theories contain nonobservational terms (e.g., attitude, intention, self-efficacy), yet predictions are tested at the observational level. To get from the nonobservable level to the observable level, auxiliary assumptions are needed. Specifically, auxiliary assumptions traverse the distance from the nonobservational terms at the theoretical level and the observational terms at the level of the empirical hypotheses.

Let us take Fishbein and Ajzen’s (1975) assumption that a person’s attitude towards a behavior should correlate with their intention. If we were to test this prediction, we would have two nonobservational terms in attitude and intention. To represent these two nonobservational terms, we would need to consider how they are represented at the observational level. Typically, these theoretical terms are represented at the observational level by check marks placed on a questionnaire. Therefore, the empirical prediction takes place by assessing the observable check marks. Because of this, it is important that a person’s unobservable attitude is indeed represented by the check marks on the attitude measures. Similarly, a person’s unobservable intention should also be represented by the check marks on the intention measures. If this does not happen and the prediction fails, this says nothing about the theory. Afterall, the theory is about the relationship between the unobservable theoretical constructs, not about the validity of observable measures. Therefore, auxiliary assumptions are necessary to test the theorized relation between unobservable attitude and unobservable intention.

As another example, imagine we wish to test Newton’s theory of gravitation. To do so, we drop a large rock and a feather at the same time, from the same height. Crucially, this is performed in an artificially created vacuum. Therefore, despite the mass of the rock being different to the mass of the feather, the two objects should fall at the same rate and contact the floor at the same time. To test this prediction, it is important to consider other assumptions not part of Newton’s theory. For example, it is important that we release the two objects at the same time, that we release the two objects from the same height, that we take a valid measure of time, etc. If we do not consider these auxiliary assumptions, then we are unable to make a valid conclusion about the theory.

Auxiliary assumptions are also relevant for evaluating the success of manipulations (Trafimow, 2012). Imagine we devise an experiment to test the theory that people feeling a sense of threat will demonstrate prejudicial behavior towards outgroup members (Stephan and Stephan, 2000). The experiment has one group read a passage of text with a threatening message and a second group read some irrelevant text. Our prediction would comprise two unobservable constructs of threat and prejudice. For the unobservable threat construct, it is important that the piece of text really does modify these perceptions. And for this to happen, participants would need to understand the piece of text, believe the piece of text, believe the text to be relevant, etc. If this is not the case, a manipulation would find it difficult to test whether a threatening manipulation leads to prejudicial behavior. Thus, the manipulation relies on important auxiliary assumptions.

Because predictions do not only rely on theoretical terms, including auxiliary assumptions necessitates syllogism 1 be updated. We can see this in syllogism 2:

### Syllogism 2

Premise 1: if the theory is true and the auxiliary assumptions are true, then the finding should be true.

Premise 2: the finding is not true.

Conclusion: therefore, either the theory is not true or at least one auxiliary assumption is not true.

As can be seen in syllogism 2, the addition of auxiliary assumptions complicates the appraisal of empirical findings. If we make a prediction and it does not come true, the empirical defeat could correctly indicate that the theory really is wrong. An alternative explanation is not that the theory is wrong but there exists at least one false auxiliary assumption attached to the prediction (Duhem, 1954; Earp and Trafimow, 2015; Meehl, 1978; Trafimow, 2009). Taking our attitude-intention example, the empirical defeat could either be because attitude does not correlate with intention or it could be that the researcher applied a poor auxiliary assumption (St Quinton et al., 2021). The relevance of auxiliary assumptions does not stop at empirical defeats, though. A prediction that comes true could indeed be because the theory is true. Alternatively, the successful prediction may instead be a consequence of a false auxiliary assumption (Trafimow, 2017). Because auxiliary assumptions have profound implications on the validity of empirical findings, it is paramount that researchers not only consider theoretical terms. Instead, researchers must also consider the auxiliary assumptions associated with the prediction (St Quinton and Trafimow, 2022; Trafimow, 2022).

The relevance of auxiliary assumptions extends beyond a single empirical finding. When undertaking replication, a finding is typically deemed successful if the study demonstrates an effect in the same direction to that of the original finding. Yet, claims about the validity of the finding can be misleading in the absence of auxiliary assumptions. If a researcher successfully replicates a study finding, whether through a direct or conceptual replication, there could be at least one false auxiliary assumption attached to the prediction. In this case, although the second study supported the findings of the first, this said nothing about the validity of the findings. Had the second researcher attended to the false auxiliary assumption, she may have found something different. Researchers attempting replications should therefore also consider the auxiliary assumptions attached to their prediction (Trafimow, 2019; Trafimow et al., 2024).

In sum, researchers must consider auxiliary assumptions when evaluating empirical findings. Low quality auxiliary assumptions can lead researchers to draw false conclusions from their data about the truth or usefulness of theories. Now that we have introduced the importance of auxiliary assumptions, we will now discuss these assumptions in the context of preregistration.

### Auxiliary assumptions and preregistration

We have briefly introduced the purpose and supposed benefits of preregistration in psychological research. We then outlined the importance of considering auxiliary assumptions when deriving predictions from theories. Specifically, we demonstrated how appraising the theoretical implications of empirical victories or defeats depends on the auxiliary assumptions associated with the prediction. Given that the validity of evaluating empirical findings depends on the appreciation of auxiliary assumptions and the purpose of preregistration is to improve the credibility of research findings, it logically follows that preregistration practices should allow for the inclusion of auxiliary assumptions in such practices. As we shall now see, the extent to which this happens can be questioned.

Preregistration has been argued to limit QRPs which, in turn, increases the validity of study findings (Nosek et al., 2018). Yet, in the same way that successful replications in the absence of auxiliary assumptions need not lead to valid findings, limiting QRPs but neglecting auxiliary assumptions may lead to the same outcome. That is, preventing cherry-picking results, *p*-hacking, HARKing, selective reporting etc., need not guarantee predictions are evaluated correctly. To illustrate, let us imagine a researcher preregisters their hypothesis *a priori*. After analysing the data, she is disappointed to find that the prediction did not come true. Of course, the researcher cannot now provide a *post hoc* hypothesis in line with the findings because, after all, she made clear her predictions in the preregistered report. Despite this preregistration benefit, she did not consider an important auxiliary assumption associated with the prediction. In this scenario, she cannot now be sure that the empirical defeat is because the theory is wrong, as she reports, or is instead due to the omitted important auxiliary assumption. Therefore, the empirical finding may still be evaluated incorrectly despite the researcher preregistering his expectations. We can see here how the credibility of a research finding need not increase in the presence of preregistration.

Although current preregistration practices do not require auxiliary assumptions, that does not mean that they are overlooked entirely. Indeed, auxiliary assumptions are likely captured inadvertently when researchers deposit details associated with their methods, hypotheses, and analysis plan. For example, the researcher may provide information about manipulation checks, the validity of construct measures, and how data will be dealt with if non-normally distributed. These are important auxiliary assumptions. However, it may not be the case that the researcher understands how the prediction depends on them. If we take the manipulation check as an example, researchers typically assume a successful manipulation if differences are found between conditions on the variable of interest. Yet, they may not consider that a manipulation check was successful due to a theoretically wrong reason. Or perhaps instead of a poor manipulation, a failed manipulation check could be due to issues with the check itself. Therefore, although some auxiliary assumptions may be captured in preregistration, this need not indicate that researchers necessarily appreciate their importance when it comes to evaluating the prediction.

If preregistration practices inadvertently account for *some* auxiliary assumptions, it logically follows that not *all* will be considered. Failure to consider a single yet important auxiliary assumption can have dramatic consequences for the prediction (Earp and Trafimow, 2015; Trafimow, 2009). For example, we mentioned earlier some auxiliary assumptions associated with a threat manipulation. In this example, participants would at least need to believe the piece of text to be threatening. Presently, there is no way of knowing whether this auxiliary assumption was considered. That is, current preregistration templates allow for many details, yet this is one likely to be evaded. And if the researcher had not considered this auxiliary assumption, an empirical defeat may not be due to a theoretical failure.

In sum, current preregistration practices could, at best, enable researchers to inadvertently account for some auxiliary assumptions associated with a prediction. However, researchers may not fully appreciate the importance of these assumptions or may not even consider the important auxiliary assumptions altogether. This is problematic when it comes to evaluating the validity of a prediction. We shall next discuss improvements to current preregistration practices.

### Improving current preregistration practices

Current preregistration practices are currently insufficient to capture auxiliary assumptions. We now offer some suggestions about how preregistration can be improved to accommodate these important assumptions before outlining some potential advantages in doing so.

It is obviously important that researchers are afforded the opportunity to state the important auxiliary assumptions associated with the prediction. To enable this, relevant space should be made available on preregistration platforms. It would be logical for room to be made after researchers provide their hypotheses. In this way, researchers would firstly state their hypotheses and then detail the auxiliary assumptions associated with the prediction. In the absence of this space, researchers could currently state the auxiliary assumptions in other sections of a preregistered report such as those labelled ‘additional’ or ‘other’ information.

Whether it is compulsory for researchers to deposit study details varies between preregistration platforms (Bakker et al., 2020; Hardwicke and Wagenmakers, 2023). Some platforms mandate some (e.g., sample size calculation, measures, hypotheses) but not all (e.g., recruitment strategy) details to be deposited. Others are far more flexible and allow the researcher to decide what to deposit. Given the importance of auxiliary assumptions, we believe that the preregistration platforms mandating certain details should also necessitate auxiliary assumptions. For those that are flexible and allow researcher freedom, it is nevertheless important that appropriate space is made available for auxiliary assumptions to be stated.

The inclusion of auxiliary assumptions in preregistration practices can yield many benefits for researchers, the review process, and psychological science more generally. Let us begin with researchers. If a researcher specifies the auxiliary assumptions associated with their prediction *a priori*, they would be better positioned to evaluate an empirical victory or defeat. Stating the conditions influencing the prediction would provide greater clarity as to whether a failed prediction is because the theory really is wrong or at least one auxiliary assumption is false. Similarly, the researcher would have a better idea if a prediction that comes true is because the theory really is true or at least one auxiliary assumption is wrong.

Another benefit for researchers is that preregistering auxiliary assumptions could lead to improvements in study quality. A researcher considering auxiliary assumptions prior to data collection may notice flaws in the study plan and subsequently amend the protocol. In addition to improving study quality, revising a study protocol before commencing with data collection would save valuable resources.

Preregistering auxiliary assumptions can be beneficial to other researchers. Knowing how a research finding was evaluated and a conclusion made can provide useful insights. To illustrate, consider Researcher 1 encounters an empirical defeat. Researcher 2 then looks over the preregistered report and finds that several relevant auxiliary assumptions were noted. However, Researcher 2 believes that an important auxiliary assumption was missed, and so decides to run the study again whilst accounting for this additional auxiliary assumption. In the case of a second empirical defeat, it might really be that the theory is wrong. However, if the new auxiliary assumption produces an empirical success, the defeat encountered by Researcher 1 may not have been a theoretical failure. Irrespective of which outcome comes true, the main point is that preregistering auxiliary assumptions can provide transparency about the conditions under which predictions do or do not come true. This transparency can, then, benefit researchers interested in the same topic.

In relation to the review process, preregistering auxiliary assumptions could prove beneficial for editors and reviewers. Because the researcher would have (hopefully) stated the relevant auxiliary assumptions associated with the prediction, these decision makers would have a better understanding as to how the prediction was evaluated. This understanding would, subsequently, better inform the decision about acceptance. If a reviewer doubts whether a single yet important auxiliary assumption was considered, the manuscript may be viewed less favorably. At the most extreme end, they may decide that the auxiliary assumption constitutes a fundamental study flaw and therefore reject the manuscript. Alternatively, the reviewer may decide to accept the manuscript if the researcher acknowledges the auxiliary assumption in the paper (i.e., limitations, future directions). A third option is the reviewer asks for additional tests to be conducted, all the while considering the auxiliary assumption. Of course, this decision would depend on many factors such as the cruciality of the auxiliary assumption, whether other auxiliary assumptions were considered, the strength of the study, etc. Yet, the fact the auxiliary assumptions were stated provides important insights into the study for the reviewer. On a positive note, a manuscript with an associated preregistered report comprising well-thought-out auxiliary assumptions should be viewed more favorably.

Perhaps the most important benefit of preregistering auxiliary assumptions is the positive impact on psychological science. Researchers would become more confident in their empirical defeats and victories and would have a greater understanding about how these conclusions were made. In turn, this confidence and understanding would increase the accuracy and credibility of study findings in psychology. Furthermore, transparency about auxiliary assumptions can also help improve the validity of replication attempts.

### Additional considerations

Including auxiliary assumptions in preregistration practices would be beneficial for many reasons. There are some additional comments and suggestions we feel worth making. First, it is important to note that the examples of auxiliary assumptions provided throughout this paper are only illustrative. Indeed, there will be many other relevant auxiliary assumptions associated with predictions in psychology.

Second, it is worth keeping in mind that the number of auxiliary assumptions will increase when more theoretical terms are added to a prediction. Auxiliary assumptions will also differ depending on the nature of research undertaken (e.g., experimental, cross-sectional, observational). For example, an experimental manipulation would need to consider the auxiliary assumptions traversing the distance from the observable manipulation to the unobservable construct. Of course, these assumptions are redundant if the study does not involve a manipulation.

Third, the responsibility of identifying relevant auxiliary assumptions to preregister lies with the researcher. This process may seem overwhelming, especially given the potentially large number of auxiliary assumptions to choose from. We have some suggestions on how this can be accomplished. First, researchers need not explicitly check each and every possible auxiliary assumption. There may be some auxiliary assumptions that the researcher is confident will be accounted for. For example, a researcher may be confident that the graduate student will enter the data correctly, or that participants will understand the questionnaire items. Therefore, instead of preregistering these auxiliary assumptions, the researcher would focus on others deemed more important. Second, although the identification and selection of auxiliary assumptions is down to the researcher, knowledge about those important to consider could be gained by consulting the literature. If a researcher is applying a particular theory, for example, then research related to the theory may highlight relevant auxiliary assumptions. For example, Fishbein and Ajzen (1975) outline auxiliary assumptions pertaining to measurement if a researcher is interested in understanding the role attitude plays in social behavior. Similarly, consulting literature covering methodological practices could reveal important auxiliary assumptions associated with a particular research design. Third, we would expect that once auxiliary assumptions become part of preregistration practices, identifying relevant ones would eventually become an easier task. Through reading the preregistration reports of those in their field, researchers would become educated about the type of auxiliary assumptions that have (or have not) and should (or should not) be considered.

Finally, researchers must note that empirical predictions not only rely on theoretical and auxiliary assumptions but necessitate the consideration of statistical and inferential assumptions (Trafimow, 2019). Statistical assumptions traverse the gap from the empirical hypothesis to the statistical hypothesis. For example, researchers in psychology typically assume a normal distribution and thus report means and standard deviations. However, most distributions are skewed (Blanca et al., 2013; Ho and Yu, 2015) and thus depend on skew normal statistics (scale, shape, and location). Yet, a sample mean and standard deviation may not correspond to the skew normal parameters of location and scale, respectively (Trafimow, 2019). In fact, there is a non-trivial probability that differences in means and differences in locations will support opposing substantive conclusions (Trafimow et al., 2023). Because of this and irrespective of good theoretical and auxiliary assumptions, an empirical finding based on assuming a normal distribution may provide a false picture when using skew normal statistics may more accurately reflect empirical reality. Therefore, researchers should attend to the statistical assumptions associated with their prediction. Inferential assumptions traverse the gap from the statistical hypothesis to the inferential hypothesis. To generalize to the population level, a researcher must consider, amongst other things, random selection, random assignment, and sample size. For example, there is no good reason to believe that findings would generalize to the population if participants were not randomly sampled. Therefore, to be confident that a sample statistic accurately reflects a corresponding population statistic, researchers must employ inferential assumptions. In sum, the evaluation of an empirical defeat or victory depends not only on theoretical and auxiliary assumptions but also statistical and inferential assumptions.

## Conclusion

Researchers in psychology are strongly encouraged to engage in preregistration practices. The assumption is that transparency will increase the credibility of study findings. However, it is important that preregistration practices consider auxiliary assumptions, especially since these assumptions play a crucial role in evaluating empirical victories and defeats. Despite a reduction in QRPs, neglecting important auxiliary assumptions may not lead to more credible findings. Therefore, if, as expected, the necessity to preregister continues to grow, preregistration practices should accommodate for the inclusion of auxiliary assumptions. This could improve the overall quality and credibility of psychological research.

## Data Availability

The original contributions presented in the study are included in the article/supplementary material, further inquiries can be directed to the corresponding author.
